# Phospholipase A_2_ Activity in Gingival Crevicular Fluid from Patients with Periodontal Disease: A possible Marker of Disease Activity

**DOI:** 10.1155/S0962935194000037

**Published:** 1994

**Authors:** H. Ishida, H. Shinohara, T. Nagata, S. Nishikawa, Y. Wakano

**Affiliations:** Department of Periodontology and Endodontology School of Dentistry Tokushima University 3 Kuramoto-cho, Tokushima Tokushima 770 Japan

## Abstract

The activity of phospholipase A_2_ in human gingival crevicular fluid (GCF) associated with periodontal disease was demonstrated. Based upon the presence or absence of bleeding on probing (BOP), which is a marker for the disease activity, there were higher levels of the enzyme activity in BOP positive, than in negative sites. When the BOP positive sites became negative after periodontal therapy, the enzyme activity decreased dramatically to almost undetectable levels. There were no significant differences between the activity before and after treatment when the BOP positive sites remained unchanged. These results suggest that the activity in GCF reflects periodontal disease conditions and that it can be used as a marker for disease activity.


Mediators of Inflammation 3, 17–21 (1994)

### Phospholipase $A_2$ activity in gingival crevicular fluid from patients with periodontal disease: a possible marker of disease activity

H. Ishida,[CA] H. Shinohara, T. Nagata, S.Nishikawa and Y. Wakano

Department of Periodontology and Endodontology, School of Dentistry, Tokushima University, 3 Kuramoto-cho, Tokushima, Tokushima 770, Japan

[CA] Corresponding Author

**THE activity of phospholipase $A_2$ in human gingival crevicular fluid (GCF) associated with periodontal disease was demonstrated. Based upon the presence or absence of bleeding on probing (BOP), which is a marker for the disease activity, there were higher levels of the enzyme activity in BOP positive, than in negative sites. When the BOP positive sites became negative after periodontal therapy, the enzyme activity decreased dramatically to almost undetectable levels. There were no significant differences between the activity before and after treatment when the BOP positive sites remained unchanged. These results suggest that the activity in GCF reflects periodontal disease conditions and that it can be used as a marker for disease activity.**

**Key words:** Disease activity, Gingival crevicular fluid, Periodontal disease, Phospholipase $A_2$

### Introduction

Phospholipase $A_2$ ($PLA_2$) is a key enzyme in the production of potent inflammatory mediators, namely prostaglandins, leukotrienes and platelet activating factor.[1] In fact, high levels of $PLA_2$ activity have been found in either the sites and/or sera of patients with various inflammatory diseases such as rheumatoid arthritis,[2,3] acute pancreatitis,[4,5] inflammatory bowel disease,[6] and septic shock.[7] Furthermore, $PLA_2$ activity in human synovial cells,[8] rat astrocytes,[9] and rabbit articular chondrocytes[10] was markedly induced in response to inflammatory cytokines such as interleukin 1 (IL-1) and tumour necrosis factor (TNF). The serum $PLA_2$ activity in some of those conditions correlates with disease activity.[6,11,12] Collectively, these results suggest that $PLA_2$ plays an important role in the process of inflammatory disease and that the determination of the activity can be a useful tool for diagnosis.

Periodontitis, an infectious disease which leads to destruction of the periodontal tissue, is a major cause of tooth loss in adults. Gingival crevicular fluid (GCF) is an exudate from periodontal tissue that contains various inflammatory mediators. For example, higher levels of $PGE_2$ and IL-1 have been detected in GCF[13,14] as well as in periodontal tissue[15,16] from patients with periodontal disease than in normal subjects. Recently, the authors characterized $PLA_2$ activity in healthy rat gingiva[17] and showed that it is regulated by inflammatory cytokines in rat gingival fibroblasts.[18,19] These results suggested that there should be $PLA_2$ activity in GCF in periodontal patients. However, there have been no reports describing $PLA_2$ activity in human periodontal sources associated with either healthy or inflamed conditions. Here, $PLA_2$ activity in GCF in patients is demonstrated and the relationship between the enzyme and periodontal disease activity is discussed.

### Material and Methods

*Patients:* Thirty-two patients ranging in age from 25 to 73 years with a mean $\pm$S.E. of 43.5 $\pm$ 1.9 years were enrolled in this study. All the patients were diagnosed as having at least several teeth with the disease and none had received any medication or periodontal therapy prior to the study. The periodontal conditions of each patient were evaluated by the depth of periodontal pocket (pocket probing), a marker of periodontal tissue destruction[20] and bleeding on probing (BOP), a marker of the active phase of the disease.[21]

*GCF sampling:* GCF was sampled from 108 sites of the 32 patients at the first visit, and in some experiments, 19 sites were selected from which to sample GCF before and after periodontal therapy. GCF sampling was performed according to the procedure of Kryshtalskyj *et al.*[22] using a 2 $\mu$l micro capillary tube (Drummond Sc. Co., USA). Briefly, after careful removal of supragingival dental plaque the tube was gently placed near the bottom of the periodontal pocket for 30 s. After sampling, the tube containing the sampled GCF was swiftly removed, the volume was measured, and the GCF in the tube was eluted into 200 $\mu$l of assay buffer containing 50 mM Tris–HCl (pH 7.4) with 10 mM

© 1994 Rapid Communications of Oxford Ltd

$CaCl_2$. The samples were stored at $-20°C$ until the assay for $PLA_2$ activity.

*Gingival tissues, blood samples and saliva:* Inflammatory gingival tissues were taken from the seven sites of seven patients immediately after collection of GCF upon pocket curettage (a periodontal therapy). The tissues were rinsed thoroughly with ice-cold isotonic solutions, weighed, then minced and homogenized in 50 mM Tris–HCl (pH 7.4) using a glass homogenizer immersed in ice-cold water, followed by centrifugation at 1 500 × *g* for 30 min. The supernatant was used as the enzyme source (the supernatant). Blood samples were collected after GCF sampling, at six sites where BOP was observed and saliva was collected from the mouths of six patients with a micro capillary of the same size as that described above.

*Enzyme assay:* The assay for $PLA_2$ activity was performed using the modified methods of Nishijima *et al.*[23] and Shakir.[24] Briefly, the assay mixtures contained 50 mM Tris–HCl (pH 7.4), 10 mM $CaCl_2$ and 10 $\mu M$ of 1-acyl-2-[1-$^{14}C$]-arachidonyl phosphatidylethanolamine (PE) (Amersham, UK) as the substrate. The incubation was initiated by adding the sample (GCF, the supernatant, blood, or saliva) to the reaction mixture and incubating at 37°C for 3 h. $PLA_2$ activity was determined by measuring the amount of [1-$^{14}C$]-arachidonic acid released from the radiolabelled PE as described previously.[17]

*Statistical analysis:* Data are expressed as means ± S.E. All data were analysed by means of the Pearson product moment correlation coefficients, the Mann–Whitney *U* test, or the Wilcoxon signed-rank test, as appropriate. The analyses were carried out on an Apple Macintosh Quadra 700 computer (Apple Computers, Cupertino, CA, USA) using Statview plus (Brain Power, Calabasas, CA, USA) software. $p < 0.05$ was considered the minimal significant value.

### Results

GCF samples taken from 108 sites of 32 untreated periodontal patients were assayed for $PLA_2$ activity (Fig. 1). About half (56 sites) of the sampled GCF had measurable $PLA_2$ activity while the other half (52 sites) had undetectable activity under our assay conditions. The levels of $PLA_2$ activity showed a wide range of total activity ranging from 2.4 to 291.0 pmol/h/sample with a mean ± S.E. of 32.4 ± 6.0.

Figure 2(A) and (B) show the relationship between $PLA_2$ activity in GCF and the GCF volume or probing depth. The volume and probing depth varied from 0.05 to 1.40 $\mu l$ with a mean ± S.E. ($n = 108$) of 0.30 ± 0.02 $\mu l$ and from 2 to 11 mm

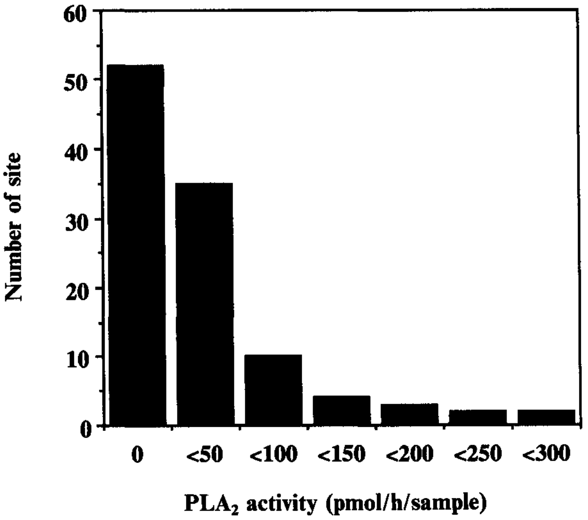

FIG. 1. Frequency histogram of $PLA_2$ activity in GCF from the sites at the first visit. The frequency distributions showed that about half of the sites examined had measurable $PLA_2$ activity, whereas the other half had no detectable activity under our assay conditions.

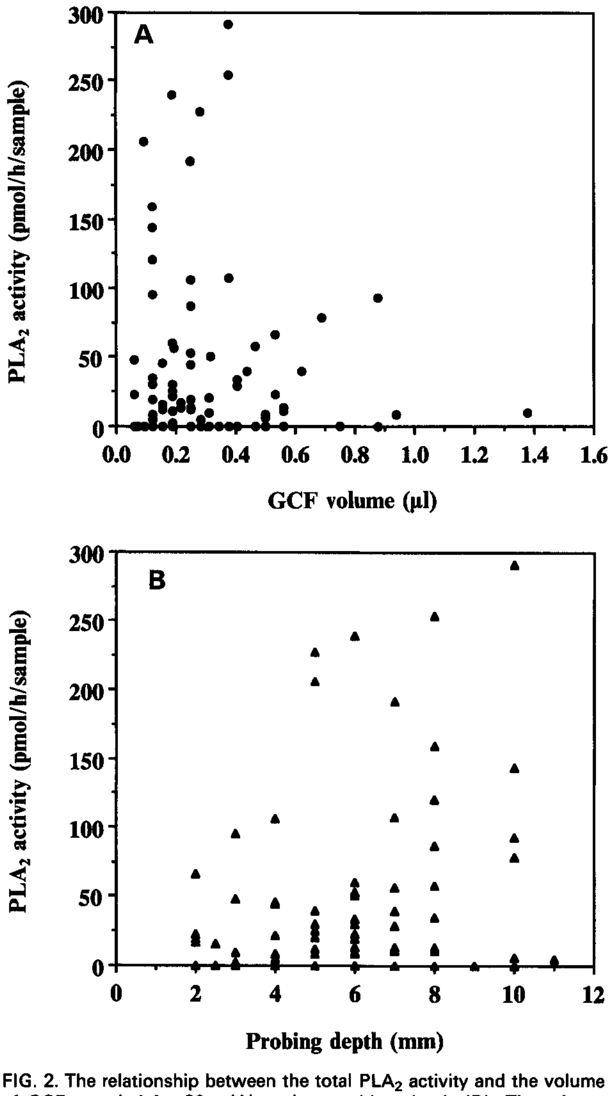

FIG. 2. The relationship between the total $PLA_2$ activity and the volume of GCF sampled for 30 s (A) and or probing depth (B). There is no significant relationship between the enzyme activity and either the volume or the probing depth. (Pearson product moment correlation coefficients.)

**Table 1.** $PLA_2$ activity in GCF in the presence or absence of bleeding on probing

| Bleeding on probing | Number of sites | $PLA_2$ activity (pmol/h/sample) (mean ± S.E.) |
|---|---|---|
| Presence | 67 | 42.0 ± 8.4 |
| Absence | 41 | 16.2 ± 4.8 |

$PLA_2$ activity in GCF is compared between two groups, one showed BOP and the other did not. ($p < 0.02$; Mann–Whitney *U* test)

with a mean ± S.E. ($n = 108$) of 5.6 ± 0.2 mm, respectively. No relationship was found between the enzyme activity and either the volume or the probing depth.

Although clinical parameters such as probing depth and the GCF volume have some limitations, bleeding upon probing (BOP) is a fairly accurate clinical sign of active disease.[21] Thus the sites were assigned to two groups by using the marker, BOP. Table 1 shows that 67 and 41 sites were in the BOP positive and negative groups respectively. The enzyme level in the BOP positive group was about 2.5-fold higher than that in the negative group.

It is possible that the high levels of the enzyme activity detected in GCF partially resulted from blood and/or saliva that might have entered the GCF at the sampling sites. $PLA_2$ levels in both saliva and sampled blood were determined as shown in Fig. 3. The mean ± S.E. of $PLA_2$ activity in GCF was 110.6 ± 41.2 pmol/h/μl ($n = 18$), whereas that in blood was 3.6 ± 3.6 pmol/h/μl. No $PLA_2$ activity was detected in saliva. These results suggested that the enzyme activity detected in GCF was only from gingival tissue around the crevice. To confirm this further, the $PLA_2$ activity in gingival tissue taken on periodontal therapy (curettage) from the seven patients was measured and compared with that in GCF of the same sites. As shown in Fig. 4, there was a highly significant correlation ($r = 0.93$) between $PLA_2$ activity in GCF and in the gingival tissue. These data indicated that the $PLA_2$ activity detected in GCF was mainly from the periodontal tissue around the sites.

The $PLA_2$ activity in GCF was determined before and after the periodontal treatment at 19 sites. The levels of $PLA_2$ activity were significantly decreased after treatment, suggesting a relationship between the enzyme activity and periodontal disease (Fig. 5). We therefore examined more precisely whether the $PLA_2$ activity in GCF reflects periodontal conditions (active phase or inactive phase). Thus, among the 19 sites, 14 where BOP was positive were selected and the activity was measured before and

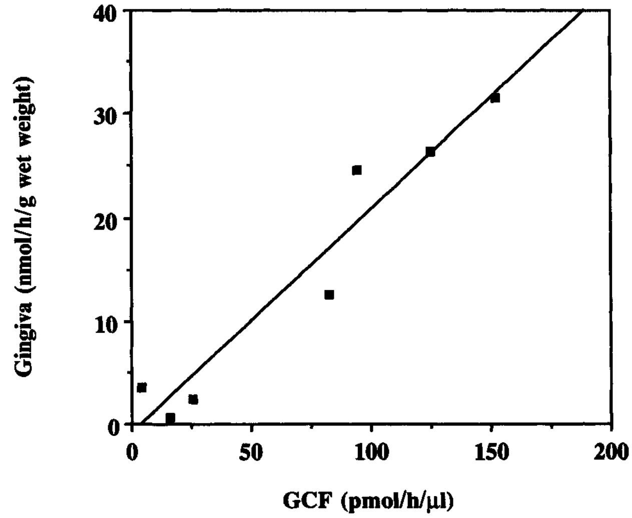

FIG. 4. Linear correlation between $PLA_2$ activity in the gingiva and GCF. ($p < 0.001$: Pearson product moment correlation coefficients.)

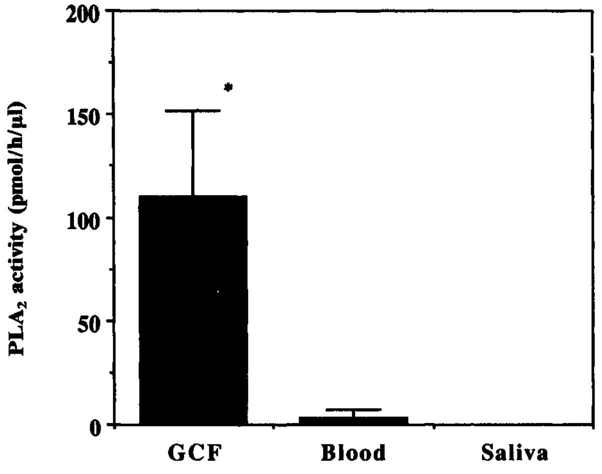

FIG. 3. $PLA_2$ activity in GCF, blood and saliva. The activity was almost negligible in the blood when compared with that of GCF and no activity was present in saliva from the same patients. ($^*p < 0.03$, compared with blood or saliva: Wilcoxon signed-rank test.)

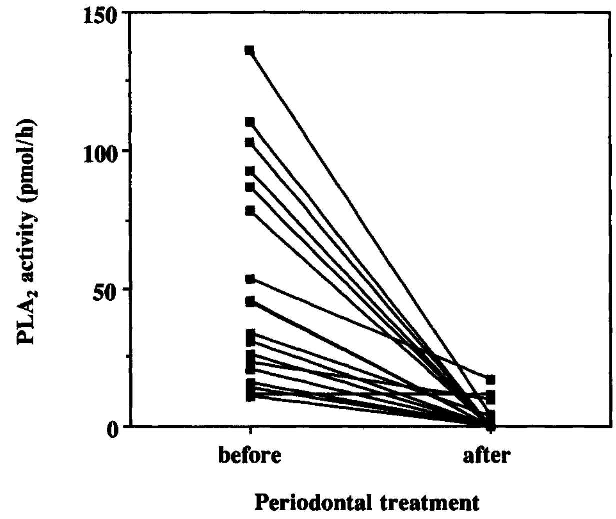

FIG. 5. Changes in $PLA_2$ activity in GCF after treatment. $PLA_2$ activity in GCF decreased significantly after treatment (62.4 ± 13.8 *vs.* 2.4 ± 1.2 pmol/h; $p < 0.001$: Wilcoxon signed-rank test.)

**Table 2.** Changes in $PLA_2$ activity in GCF after periodontal treatment

| BOP Before | BOP After | Number of sites | $PLA_2$ activity (pmol/h/sample) (mean ± S.E.) Before | $PLA_2$ activity (pmol/h/sample) (mean ± S.E.) After |
|---|---|---|---|---|
| + | + | 5 | 55.2 ± 13.2 | 5.4 ± 3.6 |
| | | N.S.[2] | N.S.[1] | |
| + | − | 9 | 78.0 ± 24.6 | 0.6 ± 0.6 |
| | | | * | |

In 14 sites where BOP were positive before periodontal treatment, the changes of $PLA_2$ activity in GCF were compared between two groups, one shows that BOP became negative after treatment and the other shows BOP remained positive. (N.S.[1]: not significant $^*p < 0.01$, Wilcoxon signed-rank test; N.S.[2]: not significant, Mann–Whitney *U* test)

after periodontal treatment (Table 2). The levels of $PLA_2$ activity in GCF at nine sites where BOP became negative after treatment, were significantly decreased to 0.8% of those before treatment. However, in five sites where BOP remained positive despite treatment, there were no significant changes in the enzyme activity.

### Discussion

The $PLA_2$ activity in GCF from patients with periodontal disease was determined. About half the GCF samples from the sites examined had detectable $PLA_2$ activity, whereas the other half had none under the present assay conditions (Fig. 1). These data suggest that there is no relationship between the enzyme activity and the disease. However, all 108 sites were divided into two groups by means of a marker for the active stage of periodontal inflammation, BOP, then the activity between the two groups was compared. The level of activity in the positive group was about 2.5 times higher than that in the negative group (Table 1). Furthermore, when 19 sites were selected from among those where $PLA_2$ activity was detected, and the activity in GCF was measured both before and after periodontal therapy, there was a significant decrease in the enzyme activity of GCF after treatment (Fig. 5). Though the total number of sites examined (five and nine) were not necessarily enough to allow interpretation of the results from Table 2, it may be of more interest that when BOP positive sites became negative after periodontal therapy, the activity markedly decreased to about 0.8% of that before therapy. In contrast, when the BOP positive sites remained unchanged even after treatment, the activity decreased to about 10%, but it was not statistically different from that before therapy (Table 2). Since periodontal inflammation typically has a relatively short active phase where tissue destruction occurs, then reaches a prolonged resting stage where apparent changes (destruction) are not observed,[25] the results in this study indicate that the activity reflects the periodontal disease stage and that it can be a marker for the active phase of this disease.

A trace of activity was found in peripheral blood that flowed from periodontal tissue into the gingival crevice (periodontal pocket) upon periodontal examination (probing) and no activity in saliva from the oral cavity was found (Fig. 3). There was a close relationship between the activity in both GCF and the periodontal tissue taken from the same sites (Fig. 4). These results indicated that the activity detected in GCF was from that produced in the tissues then secreted into the crevice.

In inflammatory disorders, such as rheumatoid arthritis,[11] acute pancreatitis[12] and Crohn's disease,[6] the activity of extracellular $PLA_2$ in the serum is correlated with disease activity and the measurement of $PLA_2$ activity has been proved to be a useful means of diagnosis. High levels of IL-1$\beta$[26] and/or TNF$\alpha$[27] in synovial fluid from patients with arthritis and in GCF from patients with periodontal disease have been reported.[14,28] Since our previous studies demonstrated that these cytokines induce $PLA_2$ activity in rat gingival fibroblasts, $PLA_2$ activity detected in GCF may result from similar events in periodontal tissue.

To the best of our knowledge, this study is the first to demonstrate the presence of $PLA_2$ activity in GCF from patients with periodontal disease. The results suggest that measuring $PLA_2$ activity in GCF will be a quantifiable marker of periodontal disease activity.

### References

1. Van den Boch H. Intracellular phospholipase A. *Biochem Biophys Acta* 1980; **607**: 191–246.
2. Vadas P, Pruzanski W. Role of extracellular phospholipase $A_2$ in inflammation. *Adv Inflam Res* 1984; **7**: 51–59.
3. Pruzanski W, Vadas P, Stefanski E, Urowitz MB. Phospholipase $A_2$ activity in sera and synovial fluids in rheumatoid arthritis and osteoarthritis. Its possible role as a proinflammatory enzyme. *J Rheumatol* 1985; **12**: 211–216.
4. Nevalainen TJ. The role of phospholipse A in acute pancreatitis. *Scand J Gastroenterol* 1980; **15**: 641–650.
5. Schmidt H, Creutzfeldt W. The possible role of phospholipase A in the pathogenesis of acute pancreatitis. *Scand J Gastroenterol* 1964; **4**: 39–48.
6. Minami T, Tojo H, Shinomura Y, Tarui S, Okamoto M. Raised serum activity of phospholipase $A_2$ immunochemically related to group II enzyme in inflammatory bowel disease: its correlation with disease activity of Crohn's disease and ulcerative colitis. *Gut* 1992; **33**: 914–921.
7. Vadas P, Pruzanski W, Stefanski E. Extracellular phospholipase $A_2$: causative agent in circulatory collapse of septic shock? *Agent and Actions* 1988; **24**: 320–325.
8. Godfrey RW, Johnson WJ, Hoffstein ST. Recombinant tumor necrosis factor and interleukin-1 both stimulate human synovial cell arachidonic acid release and phospholipid metabolism. *Biochem Biophys Res Commun* 1987; **142**: 235–241.
9. Oka S, Arita H. Inflammatory factors stimulate expression of group II phospholipase $A_2$ in rat cultured astrocytes. *J Biol Chem* 1991; **266**: 9956–9960.
10. Lyons-Giordano B, Davis GL, Galbraith W, Pratta MA, Arner EC. Interleukin-1$\beta$ stimulates phospholipase $A_2$ mRNA synthesis in rabbit articular chondrocytes. *Biochem Biophys Res Commun* 1989; **164**: 488–495.
11. Pruzanski W, Keystone EC, Sternby B, Bombardier C, Snow KM, Vadas P. Serum phospholipase $A_2$ correlates with disease activity in rheumatoid arthritis. *J Rheumatol* 1988; **15**: 1351–1355.

12. Nevalainen TJ. Phospholipase $A_2$ in acute pancreatitis. *Scand J Gastroenterol* 1988; **23**: 897–904.
13. Offenbucher S, Farr DH, Goodson JM. Measurement of prostaglandin E in crevicular fluid. *J Clin Periodontol* 1981; **8**: 359–367.
14. Charon JA, Luger TA, Mergenhagen SE, Oppenheim JJ. Increased thymocyte-activating factor in human gingival fluid during gingival inflammation. *Infect Immun* 1982; **38**: 1190–1195.
15. Goodson JM, Dewhirst FE, Brunetti A. Prostaglandin $E_2$ levels and human periodontal disease. *Prostaglandins* 1974; **6**: 81–85.
16. Jandinski JJ, Stashenko P, Feder LS, Leung CC, Peros WJ, Rynar JE, Deasy MJ. Localization of interleukin-1$\beta$ in human periodontal tissue. *J Periodontol* 1991; **62**: 36–43.
17. Shinohara H, Ishida H, Fernandez EJ, Amabe Y, Nagata T, Wakano Y. Phospholipase $A_2$ in rat gingival tissue. *J Periodont Res* 1992; **27**: 528–533.
18. Shinohara H, Amabe Y, Komatsubara T, Tojo H, Okamoto M, Wakano Y, Ishida H. Group II phospholipase $A_2$ induced by interleukin-1$\beta$ in cultured rat gingival fibroblasts. *FEBS Lett* 1992; **304**: 69–72.
19. Ishida H, Shinohara H, Amabe Y, Tojo H, Nagata T, Wakano Y. Effect of interleukin 1$\beta$, tumor necrosis factor $\alpha$ and transforming growth factor $\beta$ on group II phospholipse $A_2$ activity in rat gingival fibroblasts. *J Periodont Res* (in press).
20. Löe H, Silness J. Periodontal disease in pregnancy. I. Prevalence and severity. *Acta Odont Scand* 1963; **21**: 533–551.
21. Schluger S, Yuodelis R, Page RC, Johnson RH. *Periodonta Diseases* (second edition), Lea & Febiger, USA, 1990; 305.
22. Kryshtalskyj E, Sodek J, Ferrier JM. Correlation of collagenolytic enzymes and inhibitors in gingival crevicular fluid with clinical and microscopic changes in experimental periodontitis in the dog. *Arch Oral Biol* 1986; **31**: 21–31.
23. Nishijima J, Okamoto M, Ogawa M, Kosaki G, Yamano T. Purification and characterization of human pancreatic phospholipase $A_2$. *J Biochem* 1983; **94**: 137–147.
24. Shakir KMM. Phospholipase $A_2$ activity of post-heparin plasma: a rapid and sensitive assay and partial characterization. *Anal Biochem* 1981; **114**: 64–70.
25. Socransky SS, Haffajee AD, Goodson JM, Lindhe J. New concepts of destructive periodontal disease. *J Clin Periodontol* 1984; **11**: 21–32.
26. Fontana A, Hengertner H, Weber E, Fehr K, Grob PJ, Cohen G. Interleukin 1 activity in the synovial fluid of patients with rheumatoid arthritis. *Rheumatol Int* 1982; **2**: 49–53.
27. Ridderstad A, Abedi VM, Moller E. Cytokines in rheumatoid arthritis. *Ann Med* 1991; **23**: 219–223.
28. Rossomando EF, Kennedy JE, Hadjimichael J. Tumour necrosis factor alpha in gingival crevicular fluid as a possible indicator of periodontal disease in humans. *Arch Oral Biol* 1990; **35**: 431–434.

ACKNOWLEDGEMENT. This work was supported by a Grant-in-Aid (03454442) from the Ministry of Science, Education and Culture of Japan.

**Received 26 August 1993; accepted in revised form 21 October 1993**
